# Wireless power-up and readout from a label-free biosensor

**DOI:** 10.1007/s10544-024-00728-9

**Published:** 2025-01-10

**Authors:** Hassan Raji, Pengfei Xie, Muhammad Tayyab, Zhuolun Meng, Seyed Reza Mahmoodi, Mehdi Javanmard

**Affiliations:** 1 Department of Electrical and Computer Engineering, Rutgers University, Piscataway, NJ 08854 USA; 2 Department of Bioengineering and California Institute for Quantitative Biosciences (QB3), University of California Berkeley, Berkeley, CA 94720 USA; 3 Department of Electrical and Computer Engineering, University of Denver, Denver, CO 80208 USA

**Keywords:** Wireless Power Transfer (WPT), Biosensors, Label-free, Electrochemical Impedance Spectroscopy (EIS), Protein detection, Rheumatoid arthritis

## Abstract

Wearable and implantable biosensors have rapidly entered the fields of health and biomedicine to diagnose diseases and physiological monitoring. The use of wired medical devices causes surgical complications, which can occur when wires break, become infected, generate electrical noise, and are incompatible with implantable applications. In contrast, wireless power transfer is ideal for biosensing applications since it does not necessitate direct connections between measurement tools and sensing systems, enabling remote use of the biosensors. In addition, wireless sensors eliminate the need for a battery or energy harvester, reducing the size of the sensor. As far as we are aware, this is the first report ever describing a new method for wireless readout of a label-free electronic biosensor for detecting protein biomarkers. Our results reveal that we are able to successfully detect target protein and corresponding antibodies within this wireless setup. We are able to distinguish target protein in purified samples from a blank PBS sample as a negative control by tracking gradual changes in impedance at the input of the transmitter (*P*-value = 0.00788). We also demonstrate real-time wireless quantification of cytokines within rheumatoid arthritis patient serum samples (*P*-value = 0.00891). A Fine Gaussian Support Vector Machine is also used to differentiate protein from negative controls with the highest accuracy from a dataset of 54 experiments.

**Supplementary Information:**

The online version contains supplementary material available at 10.1007/s10544-024-00728-9.

## Introduction

Label-free biosensors are shown to be extremely useful when it comes to assessing protein levels in clinical settings (Zheng et al. 2005). Labeled detection may exhibit higher specificity in complex mixtures due to the use of tags; however, it requires additional steps and can interfere with real-time analysis, whereas label-free detection enables direct, real-time monitoring and avoids potential issues with altering the analyte's properties through labeling (Syahir et al. 2015). Label-free electronic-based technologies provide a significant advantage in ease of miniaturization and minimal use of reagents (Chung and Kim 2007; Nikfarjam et al. 2019; Hashemi et al. 2019). High salt concentrations and complicated compounds in the serum or other biological samples also impede the functionality, sensitivity, and accuracy of the electronic sensors (Sin et al. 2014). Our group has previously developed a nanowell impedance sensor that monitors the impedance across the electrodes and responds to the target protein bindings. This sensing technique can benefit from the high salt concentration in the samples in the case that a higher conductivity environment is more sensitive to the impedance altering due to the protein binding (Xie et al. 2020). Nanowell array biosensors offer significant advantages for highly sensitive, label-free detection by preventing nonspecific binding without the use of chemical or biological reagents, along with rapid and more reproducible biomarker detection due to their ability to reduce mass transfer limitations (Seo et al. 2018; Kim et al. 2008). Nevertheless, there are still several obstacles when employing nanowell impedance sensors in the clinical study. The wired system makes the experiment setup difficult to perform the in-vivo studies.

On the other hand, the wireless power transfer (WPT) system, as a novel technology, can be employed to supply power wirelessly for a variety of portable and biomedical devices (Kim et al. 2017). WPT has several advantages over traditional wired power transfer methods in the context of biosensor applications. One key advantage is increased mobility (Kilinc et al. 2015), which allows biosensors to operate without the constraints of physical cables, enabling users to move freely and enhancing the practicality of wearable and implantable devices. Proper performance of inductive coupling WPT is sensitive to coil misalignment, so the system must be designed to minimize misalignment and maintain the relative positioning of the components for consistent power transfer when the patient is mobile, without the need to disconnect wires, while still allowing continuous sensing capabilities. By eliminating the need for physical wires, biosensors can be more easily worn or carried by users, allowing for greater freedom of movement and making them more suitable for use in a variety of settings which is important in biomedical contexts where mobility is essential (Salim and Lim 2019). The overall reliability and durability are also improved. Without the need for physical connections, there is less risk of damage or interruption to the power supply, which can be critical in certain applications such as medical diagnostic devices (Ferguson and David Redish 2011). This can also reduce maintenance requirements and increase the overall lifespan of the biosensor (Haerinia and Shadid 2020). In addition, WPT can also reduce the cost and complexity of biosensors, making them more accessible and affordable for a wider range of users (Agoulmine et al. 2012). By eliminating the need for physical power connections, these devices can be simpler, less expensive, and more compact to manufacture, making them more appealing for both commercial and consumer applications. The same drawbacks as surgery-based medical treatments, including the risk of complications during the procedure, a lengthy recovery period, and a significant financial burden, are not encountered with wireless biosensors (Li et al. 2021). In addition to eliminating wired power connections, this fundamental technology offers a longer life span in comparison to batteries, and the risk of serious health hazards for biomedical applications is eliminated (Kim et al. 2017). These advantages of wireless sensors allow them to be used for a range of applications, including disease diagnosis, monitoring, tracking, and recording the vital signs of individuals (Li et al. 2021; Koydemir and Ozcan 2018). In this respect, inductive coupling WPT provides a low input voltage and also allows the non-invasive collection of data (Shobaki et al. 2013). Kim et al*.* used WPT to power their glucose sensor at the receiver side and then measured the change in glucose levels through the current (Kim et al. 2018). In another study, Luo et al*.* (2013) proposed a pressure sensor that works based on resonance. A variable capacitor is formed by two electrodes forming a sensing cavity which is connected to an inductor on the receiver side. A measurement of the resonance frequency on the transmitter side led to the measurement of the change in the capacitor gap and, therefore, the changes in pressure. Gao et al*.* proposed an RFID-based microwave biosensor for detecting glucose levels in solutions, demonstrating its potential for diabetes monitoring by showing how the resonance frequency of the squared spiral tag changes with varying glucose concentrations (Gao et al. 2021). Tanguy et al*.* developed a low-cost, wireless biosensor for *in situ* food quality monitoring by modifying a passive RFID tag with a sensing element that included conductive nanofillers/particles, a binding agent, and a polymer matrix, then exposing the tags to a food spoilage biomarker and monitoring their response over time using a network analyzer (Tanguy et al. 2015).

Besides, a biomarker refers to a substance in an organism whose presence indicates a particular phenomenon, such as disease, infection, or environmental exposure (Califf 2018). Measurements of exposures or risk factors are more sensitive and specific when biomarkers are used (Madhavan et al. 2015). The use of biomarkers to determine disease onset, progression, and treatment effectiveness is currently commonplace (Ciesla et al. 2011; Ponti et al. 2020; Scaramuzzi et al. 2019; Meng et al. 2023). There are a multitude of uses for proteins as biomarkers, including early detection of cancer, discovery of vaccines, and even serological assays to determine exposure to infectious diseases such as COVID-19 (Chikkaveeraiah et al. 2012; Ramachandran et al. 2008; Kermali et al. 2020). High mobility group box protein 1 (HMGB1) is a nuclear protein that binds DNA and increases access to transcription factors. It also contributes to the production of tumor-necrosis factor (TNF), interleukin-6 (IL-6), and interferon-γ (Lotze and Tracey 2005). Recently, there has been considerable attention on HMGB1 and its role as a biomarker in traumatic brain injury (TBI), neuroinflammatory conditions, epileptogenesis, and cognitive impairments (Paudel et al. 2018). Furthermore, an important inflammatory and autoimmune disease is Rheumatoid arthritis (RA) which the long-term effects of RA may include physical and work disability, reduced quality of life, and increased mortality (Xu and Qing 2021). An infection caused by this disease results in inflammation in the affected parts of the body as the immune system attacks healthy cells by mistake (https://www.cdc.gov/arthritis/basics/rheumatoid-arthritis.html). TNF-α is highly elevated in RA patients, and it appears to interfere with the mechanism controlling the suppressive function of Regulatory T cells (Tregs), so it can be used as a biomarker of this disease (Farrugia and Baron 2016).

To address these difficulties of wired measurements and due to the advantages of having a wireless sensor, we have developed a wireless power transfer nanowell impedance sensor to detect protein biomarkers. In order to enhance the robustness of our technology and guarantee the safety and reliability of microfabricated alternatives in future implantable devices, we initiate the study by using manually wound coils. This approach enables a comprehensive analysis of the wireless biosensor's ability to distinguish between controls and antibodies, which is crucial for developing a downsized implantable biosensor. While our study primarily focuses on this capability, it also provides preliminary insights into compatibility and higher frequency response, informing future optimization efforts. While we use HMGB1 as a proof-of-concept, we emphasize that this platform is applicable to a wide array of protein biomarkers. In order to better demonstrate the capabilities of the setup, we also test it with serum samples from RA patients to detect cytokines. We employ wireless resonant inductive coupling to measure the protein immobilization on the surface of the electrodes in a nanowell. In this biosensor, the protein immobilization to antibodies is translated into a change in the output voltage of the lock-in-amplifier on the transmitter side, which can be detected remotely. The use of wireless power-up allows performing measurements without the need to directly connect the sensor to measurement tools and is a major step for the development and miniaturization of the system.

## Theory

WPT based on inductive coupling typically uses magnetic fields to transmit near-field signals. Several parameters influence the transfer of power in this type of WPT, such as the relative location of the coils, frequency, current excitation, and coil geometry, which affect inductance. As part of inductive coupling, fundamental laws of physics are utilized, and by integrating the magnetic field generated by the current distribution within coils, Biot-Savart's Law can be used to determine the magnetic field as described in the following equation (Mou and Sun 2015):

1 $$\overrightarrow{\mathbf{B}}=\frac{{\mu }_{0}}{4\pi }\oint \frac{Id\overrightarrow{\mathbf{l}}\times \overrightarrow{\mathbf{r}}}{{\left|\overrightarrow{\mathbf{r}}\right|}^{3}}$$

In this equation, $\overrightarrow{\mathbf{r}}$, ${\mu }_{0}$, and $d\overrightarrow{\mathbf{l}}$ respectively denote the full displacement vector from the wire element, the permeability of free space, the current in the transmitter coil, and the length of the differential element of the wire. Specifically, when the distance between coils is relatively small in comparison to the gap's cross-section (Muhlethaler et al. 2011), and assuming a uniform flux density distribution in the gap, the gap reluctance can be calculated as follows:

2 $${R}_{g}=\frac{{l}_{g}}{\mu {A}_{g}}$$

In this equation, $\mu$ refers to permeability, ${A}_{g}$ denotes cross-section of the air gap, and ${l}_{g}$ is the air gap length.

3 $${V}_{ind}=-\frac{\partial }{\partial \text{t}}\oint \overrightarrow{\mathbf{B}}.d\overrightarrow{\mathbf{S}}$$

The efficiency of power transmission is higher when the transmitter coil and the receiver coil are close to each other. There is resonance coupling when the resonance frequencies of transmitters and receivers overlap. A resonant inductive coupling system is created by capacitors and inductors on either side of the circuit, thus enhancing transmission efficiency. In this study, coils are designed in a way that combines their inductance with the capacitance of their sensors to yield a resonant frequency that is the same on both sides:

4 $${L}_{r}=\frac{1}{{\omega }^{2}{C}_{s}}$$

In the above equation, ${L}_{r}$ is the receiver’s inductance and ${C}_{s}$ is the sensor’s capacitance at the resonance frequency and $\omega$ is the secondary’s resonance frequency similar to the transmitter’s resonance frequency. The wireless setup uses coil inductances that are designed in such a way that both sides have the same resonance frequency. The frequency of 10.5 MHz was chosen based on the fabrication consideration of the ultimate miniaturized sensor to be in the range of higher sensitivity for nanowell array and also on the frequency of the measurement tools available. The capacitance of the sensor was measured for this frequency, from which the coil inductance of 7.7 mH was obtained.

## Materials and methods

### Sensor fabrication

Oxygen plasma is initially used to clean a fused silica wafer (University Wafer, South Boston, MA, USA), and then the photoresist is patterned using photolithography. Next, using E-beam evaporation and patterning by lift-off, a metal layer including 5 nm Chromium and 100 nm Gold is deposited on the substrate. The deposition of the 5 nm chromium layer enhances the adhesion of the gold layer to the substrate. The above-mentioned steps result in one of the electrodes (The bottom electrode).

A 40 nm aluminum-oxide interelectrode insulator is deposited on top of the patterned layer using Atomic Layer Deposition (ALD). The same method is used to manufacture the Top electrode, connection pads, and interconnecting lines between the electrodes and pads. Next, a photoresist layer is spin-coated, and an array of micron-sized wells is patterned on top of the overlapping part of the bottom and top electrodes. Afterward, multiple wet etching processes are performed to etch the oxide layer on top of the top electrode, the gold layer, and the chromium layer. Finally, the oxide layer between the top and bottom electrodes is also etched. This is done sequentially using buffered oxide etchant, gold etchant, chromium etchant, and buffered oxide etchant. Figure 1 in the supplementary materials illustrates the etching step of the fabrication process. Finally, the photoresist is stripped off, and a mask is employed for patterning the photoresist to etch off alumina in parts of the sensor where there is no sensing region. The resulting product is the sensor containing two overlapping electrodes. A 5-mm round-shaped fluidic cell made up of PDMS is bonded on top of the array of nanowell, and connection pads are wire-bonded.

### Wireless setup

**Fig. 1:**
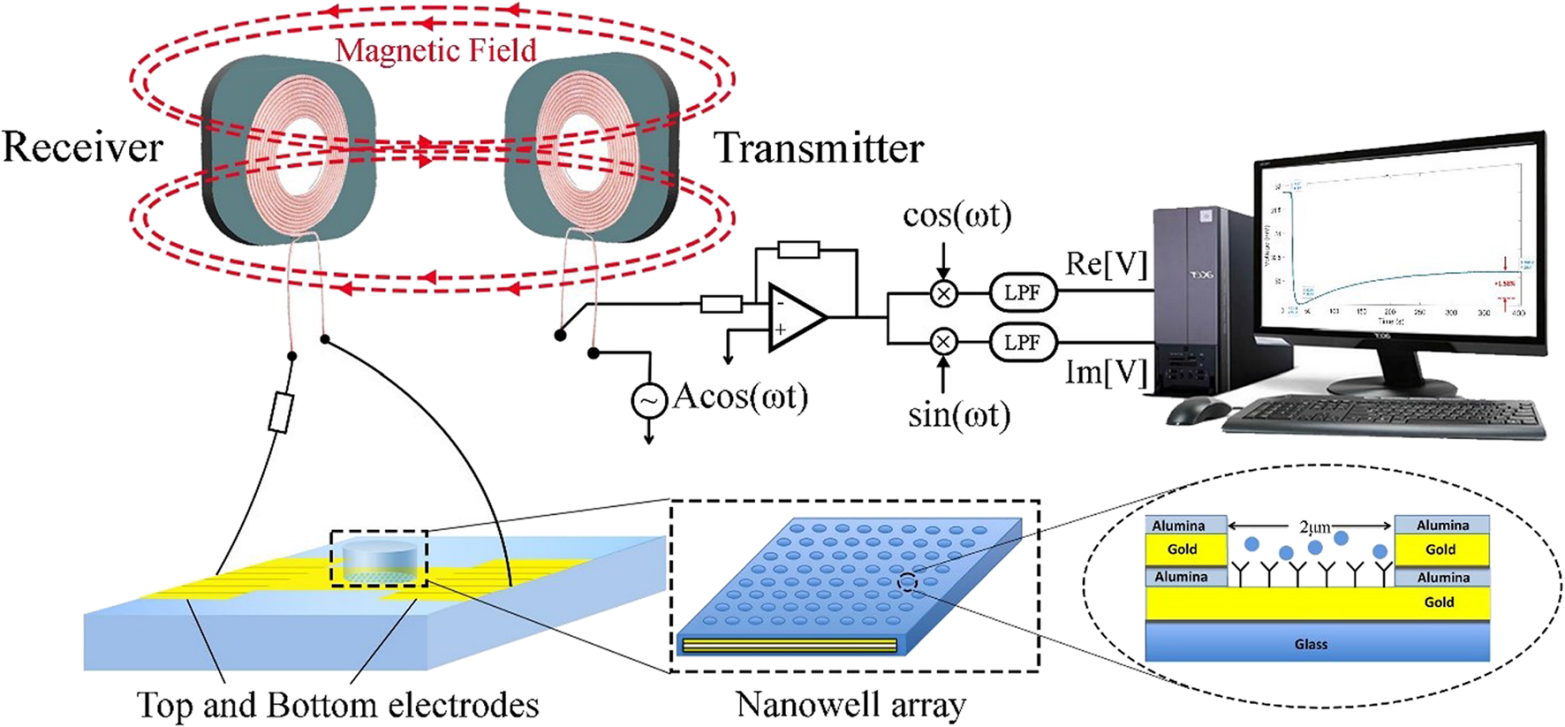
The schematic of the wireless power transfer system

The nano-well sensor includes two electrodes measuring impedance between which there is a thin dielectric oxide layer (Raji et al. 2021). There is a conductive path in this biosensor between the two overlapping electrodes (Raji et al. 2023). Real-time measurement of impedance is carried out using the two electrodes. The electrodes are in an array format, and they enable us to probe antibody immobilization inside the nano-wells. A schematic of the wireless protein biosensor setup is shown in Fig. 1.

The sensing setup consists of two inductively coupled primary (transmitter) and secondary (receiver) coils. The nano-well sensor is on the receiver side in series with the receiver coil and a 26-Ω resistor. There is no direct connection between the receiver and transmitter side. A 500-Ω resistor, a transmitter coil, and a lock-in-amplifier (Zurich Instruments HF2IS, Zurich, SI) form the transmitter side, all in series. Both primary coil and secondary coil have 35 turns that are aligned while the wire diameter is 0.6 mm and the cross-section area is 2 cm^2^. Furthermore, the entire wireless setup can be compactly housed within a 3 cm^3^ space.

When the impedance inside the nano-wells changes, this change can be measured using the Impedance Spectroscope through the lock-in-amplifier. The AC excitation source of this setup is set to 400 mV at a frequency close to the resonance frequency of the system (10.5 MHz). Any change in the impedance of the nano-well sensor can be tracked by measuring the change in the equivalent impedance seen from the transmitter. In the wireless setup, one side of the 500-Ω resistor is connected to one side of the primary coil, and another side is connected to the source. The other side of the coil is connected to the input of the lock-in-amplifier in which the real and imaginary parts of the impedance are calculated in real-time. For the lock-in-amplifier, the gain is set to 1 k, the sampling rate is 225 samples/s, and the bandwidth is 2 Hz.

## Results

### Detection of target protein in purified samples

By connecting the wireless setup to an Impedance Analyzer (IA) (Keysight Technologies E4990A IA, CA, USA), the impedance of the system can be measured in a wide range of frequencies between 20 Hz to 20 MHz.

**Fig. 2:**
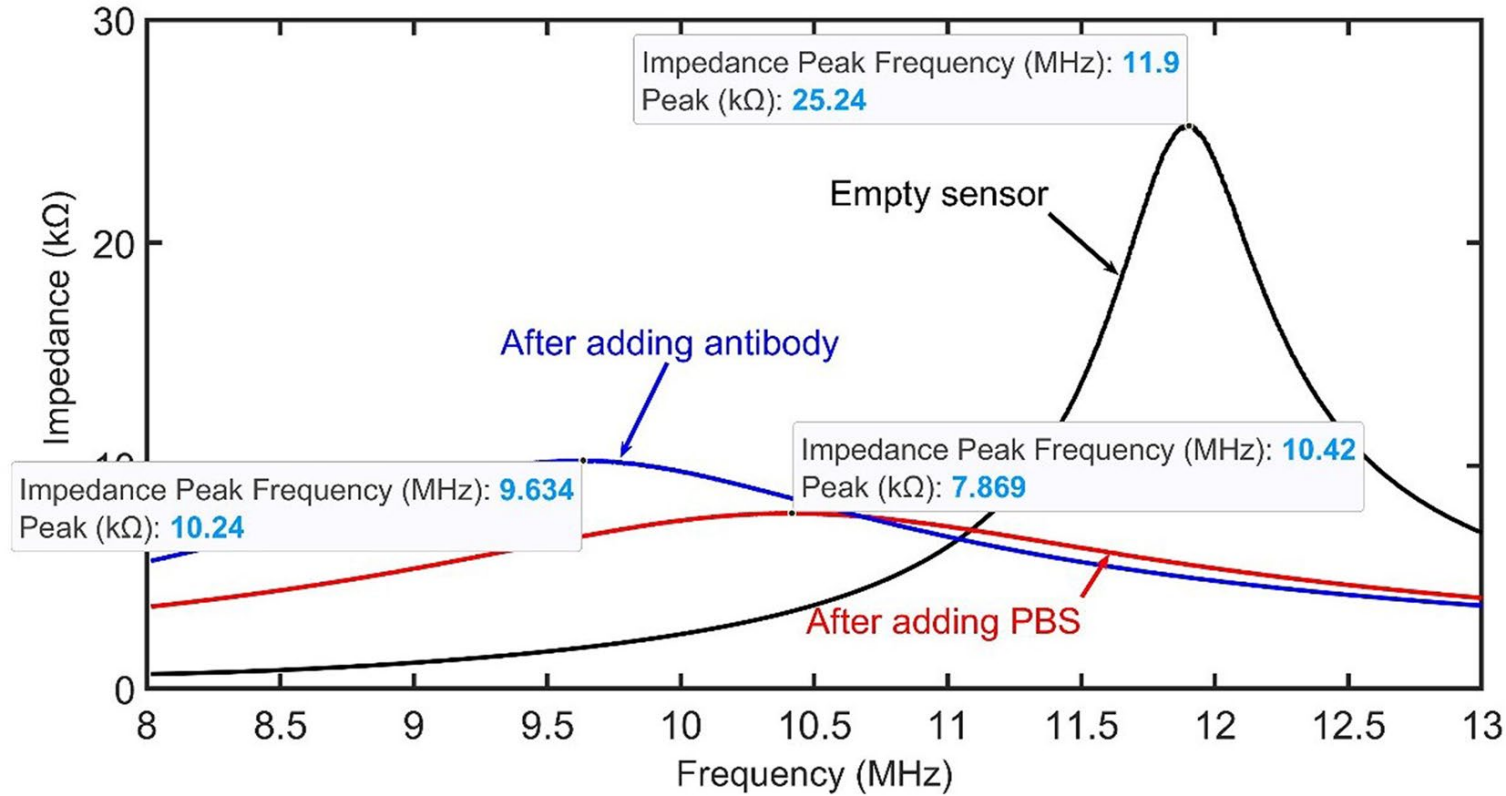
Impedance spectrum of the wireless system measured by IA

The results indicate that the maximum absolute value of the impedance occurs at 11.9 MHz when the sensor is empty (See Fig. 2). By adding phosphate buffer saline (PBS) to the sensor, this frequency shifts to 10.42 MHz, and equivalent impedance also changes greatly to less than half of the previous value when the sensor is empty. This change in the impedance spectrum is mainly because of the change in the impedance of the sensor in the secondary.

Furthermore, when adding the antibody, there is a shift in the peak and the corresponding frequency, which is in line with the theory. The main objective is to track the impedance changes while adding target protein and negative control, and to see if these two can be differentiated from one another. One way to identify the step of the experiment is to observe the impedance spectrum while adding different reagents to the sensor. A second method is to use lock-in amplification to lock-in to a single frequency and then perform measurements over that specific frequency (10.5 MHz).

The experiment is performed in triplicate and step by step in a way that each step lasts for 10 minutes. Figure 3 of the supplementary materials shows the various steps of the experimental procedure. Reagents are added to the fluidic cell. Recombinant Human HMGB1 as the target biomarker and Human HMGB1/HMG-1 antibody were commercially purchased (R & D Systems). The antibody and target biomarker are suspended in PBS, and the corresponding solutions are prepared with a concentration of 20 µM and 4 pM, respectively. Reagents are sequentially added manually to the sensor, and the complex impedance is monitored in real-time. It is essential to capture impedance changes occurring in the wireless system in real-time when occurring biological binding events. The procedure for one of the experiments is explained thereunder, but for the other two experiments, the same protocol is employed, and the data is shown in Fig. 5 of the supplementary materials.

**Fig. 3:**
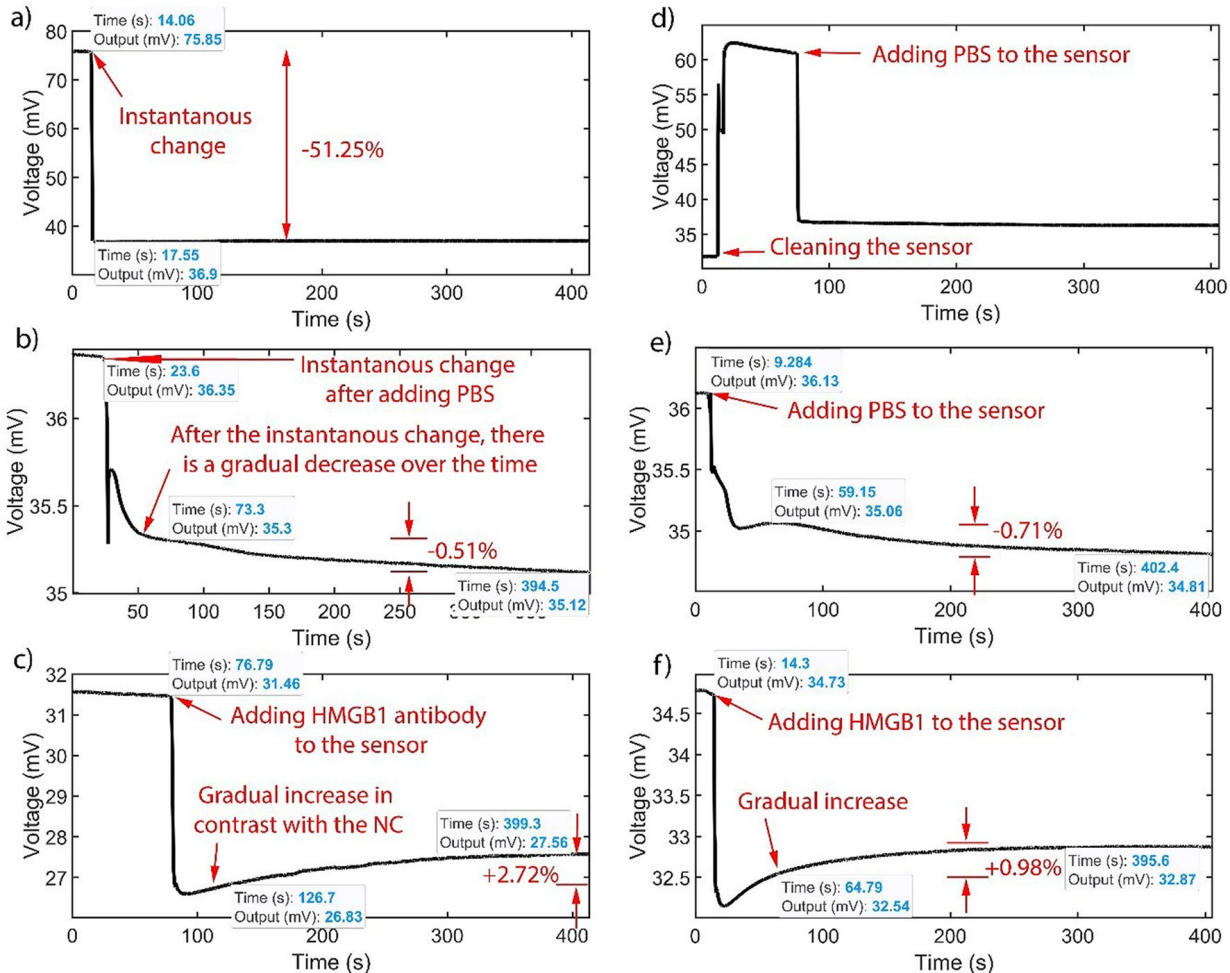
Measurements that are performed in six steps. These steps include: **a** adding 10 µl PBS to the dry sensor, **b** adding 1 µl negative control blank PBS sample, **c** adding 5 µl of HMGB1 antibody solution to the sensor. **d** Then, the sample is taken out of the fluidic cell and it is refilled with a 10 µl PBS sample. **e** Another 1 µl blank PBS sample is added to the sensor as the negative control. **f** Finally, 3 µl of HMGB1 solution is added to the sensor

The experiment starts with filling the sensor with 10 µl of Phosphate Buffer Saline (PBS) buffer. The output of the lock-in-amplifier that is connected to the primary side is shown in Fig. 3a. The experiments indicate that the output voltage decreases to almost half of the original value when the sensor is empty.

The equivalent impedance that is seen from the lock-in-amplifier measures the change in the net impedance. The absolute value of the impedance inside the sensor changes dramatically in this step so that the net impedance that is dependent on the sensor’s impedance also changes noticeably. As PBS buffer has high conductivity, the impedance in the sensor changes greatly, which is in line with the expectations. As a result, the current inputted to the lock-in-amplifier, and consequently, the output voltage, sees this abrupt change. This instantaneous change refers to the change in the baseline that occurs immediately after adding reagents to the sensor. For example, in Fig. 3a, at t = 14.06 (s), there is a significant downward change in the baseline of 51.25%. This percentage change is calculated by taking the difference between the output voltage value after the change (t = 17.55 (s)) and the output voltage value before the instantaneous change t = 14.06 (s), then dividing it by the latter value. The result of the three experiments indicates that the average change is −54.433%, with a standard deviation (SD) of 3.37.

**Fig. 4:**
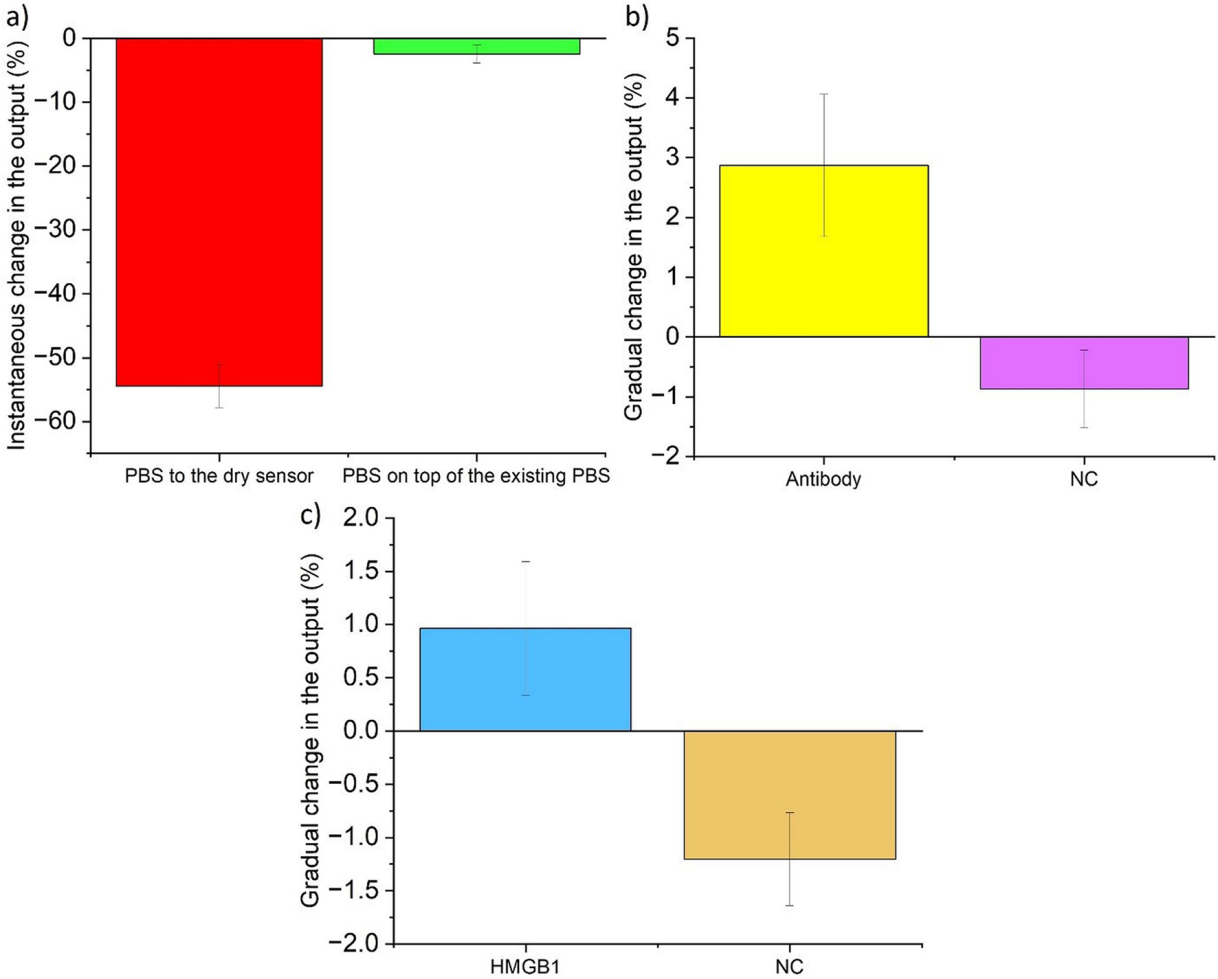
Instantaneous and gradual change of the output in different steps of the experiment. **a** Comparison of instantaneous output changes (%), with error bars, for adding PBS to a dry sensor and to a sensor with existing PBS as negative control (NC). **b** Gradual change in the output with error bars (%) for antibody and negative control (NC). **c** Gradual change in the output with error bars (%) for HMGB1 and negative control (NC). The bars indicate the average output change across the three experiments, while the error bars represent the standard deviation from the average

1 µl of blank PBS sample as a negative control is added to the sensor. As it is shown in Fig. 3b, there is a small amount of change in the negative direction in the baseline. The instantaneous change in the baseline is much smaller than what was measured in the previous step. The average of this instantaneous change for the negative control in the baseline is just −2.43%, with an SD of 1.42 for the three experiments. A comparison between the instantaneous change by adding PBS to the dry sensor and adding PBS on top of the existing PBS in the sensor as a negative control is shown in Fig. 4a.

Then, 5 µl of HMGB1 human antibody is added to the well, and antibodies are immobilized to the surface of the gold electrodes. By adding this sample, in contrast to the negative control, after the instantaneous change in the baseline, there is a gradual increase in the output voltage (See Fig. 3c). We also observed such an exponential pattern in our wired sensor (Xie et al. 2020). When the antibodies are present on the surface of the electrodes, this leads to the occlusion of ionic current, which passes between the electrodes, and the resistance also changes.

The gradual change observed for the antibody corresponds to an exponential shift in the baseline following the instantaneous change previously discussed. This behavior aligns with the time-dependent nature of antigen–antibody binding. Comparing this gradual change to that of the negative control helps eliminate the possibility of non-specific binding to the sensor surface. To ensure consistent measurement of the gradual change across all experiments, the percentage change is calculated by finding the difference between the output voltage at 400 s after recording the data and the output voltage at 50 s after sample addition. This difference is then divided by the output voltage at 50 s after sample addition. By comparing the voltage drift of the negative control step to the gradual voltage change observed in the antibody step, we can identify the differences between the two steps in this respect. The average of gradual changes for the negative control and antibody steps are −0.87% and + 2.87%, with an SD of 0.65 and 1.19 (Fig. 4b). Furthermore, the p-value between these two sets of data is 0.00891, indicating a statistically significant difference. This p-value was calculated using a one-way ANOVA to assess the significance of the output changes between the control and antibody/protein groups. This method evaluates whether there are significant differences in the means of the two groups, helping to determine the reliability of the observed changes. Besides, the saturation occurs between 10 to 15 min after adding the antibody solution to the well. Next, the extra antibodies are removed from the sensor, and 10 µl of PBS is added to the well (See Fig. 3d).

A subsequent step is adding another 1 µl of negative control on top of the solution that is already in the well (See Fig. 3e). For the 3 negative control experiments, the abrupt change in the signal baseline is 1.74% in the negative direction with an SD of 1.02 on average.

Finally, 3 µl of HMGB1 solution is added to the fluidic cell, which leads to a gradual exponential increase in the output voltage, the same as the third step, which is relevant to antibody absorption (See Fig. 3f). A similar exponential change is observed in the wired measurements for both the antibody and protein steps for the wired sensor (Xie et al. 2020). The presence of the target protein on the electrode surface obstructs the ionic current between the electrodes, resulting in a time-dependent change in resistance, which can be measured by monitoring the gradual change. Figure 4c illustrates the average gradual change for both protein and the negative control with error bars. This average change is + 0.96% for HMGB1 in the positive direction with an SD of 0.62. Yet, this average change for the negative control was 1.2% in the negative direction with an SD of 0.43. The P-value between these two sets of negative control and HMGB1 for the three experiments is 0.00788. The next section demonstrates real-time, wireless, label-free measurement of cytokines in serum samples within ten minutes.

Another set of experiments was designed to further validate the readings using the nanowell array to extract the standard titration curve for CXCL-5. The results of this study are shown in Fig. 6 of the supplementary materials, which further highlight the sensitivity and effectiveness of biomarker detection using this method in our wireless setup.

### Wireless measurement with patient sample

In the previous section, proteins were suspended in PBS. After demonstrating sensor performance in purified samples, we move on to characterizing sensor performance in spiked serum samples. As a way to represent the strengths of the current sensor setup, we use serum samples taken from patients with rheumatoid arthritis.

**Fig. 5:**
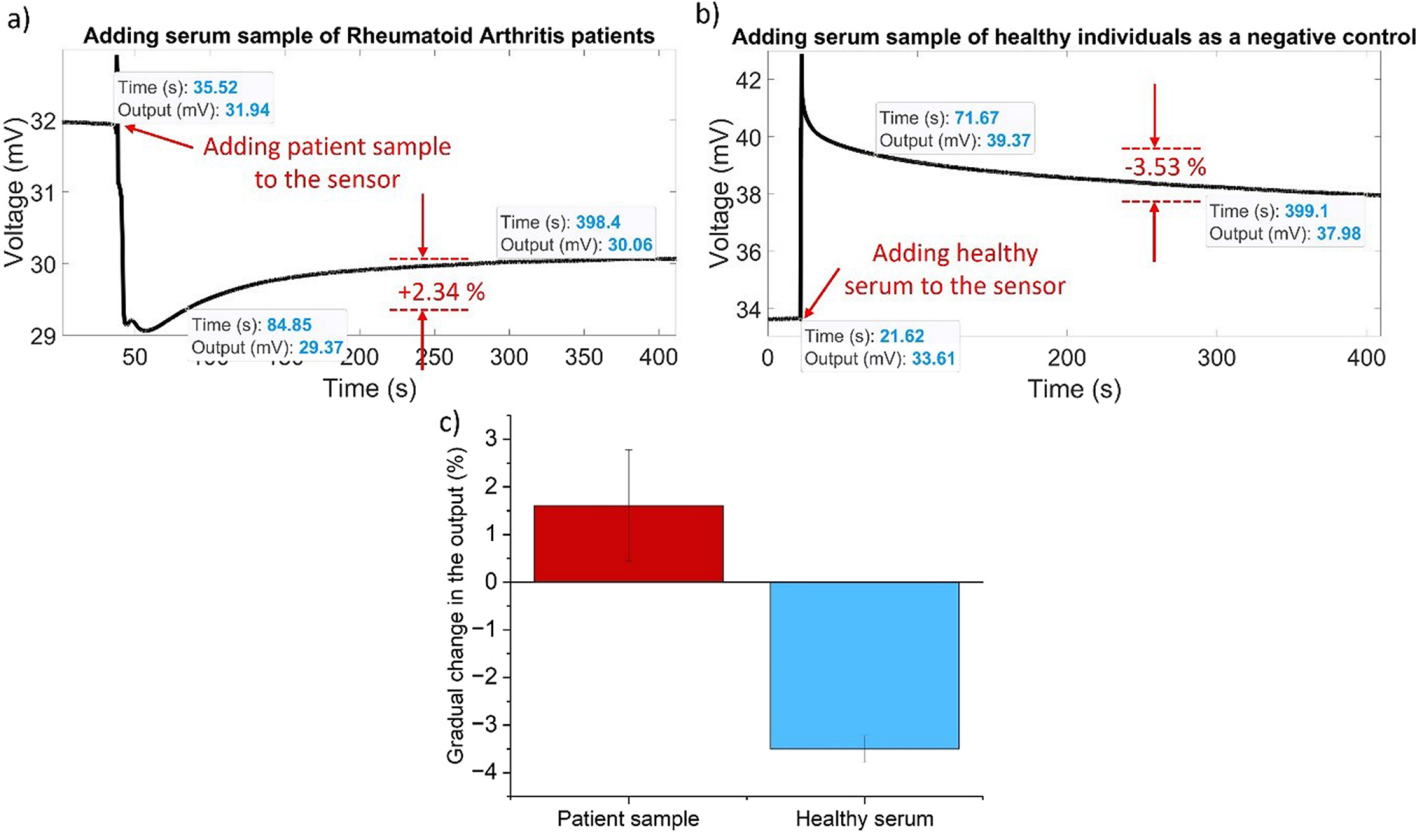
Gradual change in the output (%) after adding **a**) patient sample with high concentration of TNF-α and **b**) healthy serum as a negative control (NC). **c** Gradual change in the output with error bars (%) for serum samples from RA patients with high level of TNF-α healthy serum samples as negative control (NC). The bars display the mean output change from the three experiments, whereas the error bars illustrate the standard deviation from this mean

The procedure for preparing the sensors for the addition of target samples and negative control experiments is the same as that shown in Fig. 3 in the manuscript and Fig. 5 in the supplementary materials for all experiments. The set of experiments is also performed in triplicate to ensure that the wireless setup provides repeatable measurements. Figure 5a illustrates how the characteristic exponential decay (progress toward the positive direction) is observed over a period of minutes when the target protein (TNF-α) is present in the RA patient serum sample with a high concentration of TNF-α (120 pg/ml). However, in the case of the negative control with healthy serum containing a very low concentration of TNF-α (less than 10 pg/ml) which includes non-target proteins, the gradual change is similar to what is observed with purified samples, and the gradual direction of change is more towards the negative side in comparison to the samples containing TNF-α (See Fig. 5b).

A comparison is made between the drift in the voltage of the negative control and the gradual change in the voltage of the patient sample in this triplicate experiment. The average of the gradual changes between the negative control and patient samples addition steps are −3.5% and + 1.61% with SDs of 0.28 and 1.17. In addition, the p-value between these two sets of data is 0.00183.

### Automatic detection of target proteins and negative control using machine learning

**Fig. 6:**
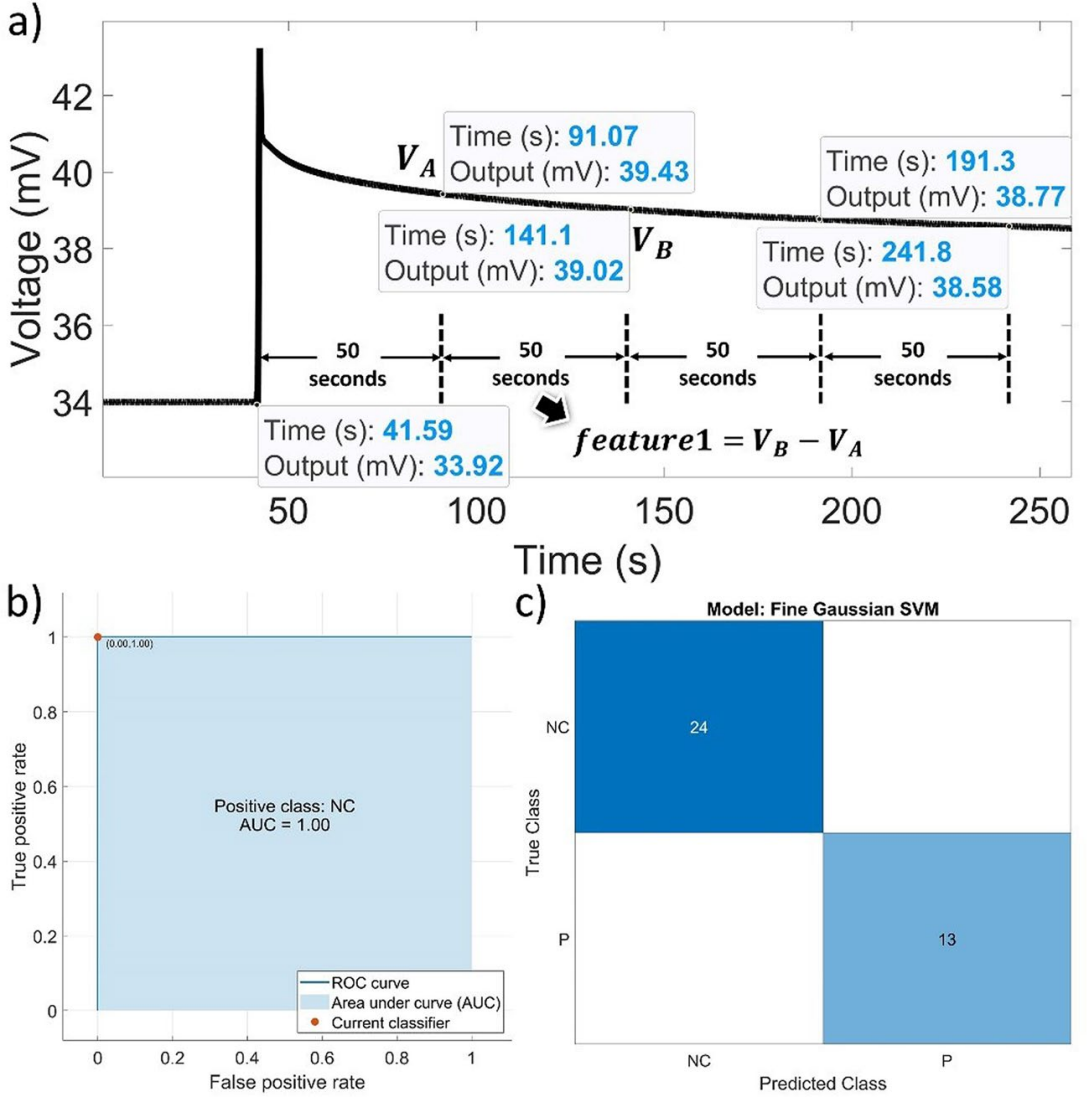
**a** Extraction of features from the output signal of the lock-in-amplifier. **b** AOC curve and **c**) confusion matrix using Fine Gaussian SVM Model

Machine learning can help identify trends in biosensors' output data and provide understandable and fast measurements. Applying machine learning to the rapidly generated large datasets from biosensors minimizes the errors that can arise from manual analysis by experienced users. This leads to faster processing time and improves sample-to-answer time as delays in processing by a person are eliminated (Raji et al. 2022). In this regard, automated detection of proteins and negative controls is carried out using machine learning techniques available in MATLAB's Machine Learning Toolbox. In this respect, a dataset is randomly gathered from 54 experiments with protein and negative control, ensuring that the selection is not biased or selective. The dataset is split into 70% as training and 30% as testing datasets through a random partitioning process. Additionally, 33.3% and 66.7% are, respectively, portions of protein and negative control data in all the datasets. After careful observation of the response of the sensor to target protein and negative control, it is observed that both NC and protein data include an instant change and a gradual change. Four features are defined in a way that the output voltage is compared in every 50 s starting 50 s after the instantaneous change. Figure 6a shows the extraction of features from the value of the output signal of the lock-in-amplifier. To illustrate, the first feature is extracted by subtracting the values of output at 100 s and 50 s after the instantaneous change (See Fig. 6a).

Both train and test data are imported into Matlab after the model is trained, and the model determines whether each row represents NC or protein. Both the training and test datasets showed that differentiation between NC and protein could be achieved with 100% accuracy using Fine Gaussian Support Vector Machines, among the techniques studied within the machine learning toolbox. The Fine Gaussian Support Vector Machine (SVM) is selected due to its robust ability to handle complex, non-linear data patterns by utilizing a Gaussian (or Radial Basis Function) kernel. This kernel maps the input data into a higher-dimensional space where linear separation becomes feasible, allowing the model to effectively capture subtle variations and distinctions between protein and negative control signals in our biosensor dataset. The Gaussian SVM’s sensitivity to intricate data structures makes it particularly well-suited for this classification task, where the differences between classes are nuanced. We also tested other machine learning models, including decision trees and k-nearest neighbors, but the Fine Gaussian SVM achieved the highest accuracy for our data distribution, making it the optimal choice for this study. The AOC curve and confusion matrix are shown in Fig. 6b and c.

## Conclusion

We performed the fabrication and implementation of a novel inductively coupled nanowell array impedance sensor for detecting target proteins in a label-free manner. Two opposing electrodes include an overlapping region which is separated by an aluminum oxide layer and contains several holes exposing the solution to the bottom electrode. This sensing modality utilizes an array of nano-wells that are functionalized with antibodies. They are embedded with electrodes to detect changes in ionic resistance when there is a binding of the target protein to the corresponding antibody inside the wells. The sensor is connected to a resonant inductive coupling wireless power transfer system, which translates the change in the impedance of the sensor to the equivalent impedance seen from the transmitter. We monitor and record the impedance from the transmitter with no direct electrical connection to the sensor. The results reveal that the binding of the target protein, HMGB1, can be tracked using our wireless setup, which can be differentiated from the negative control blank PBS sample distinctively. The data shows that after adding protein to the sensor, the output voltage of the lock-in-amplifier changes by about 0.96% in the positive direction. However, this change is 1.2% in the negative direction for the negative control. We also compare the real-time measurement of cytokines in serum samples of rheumatoid arthritis patients with serum samples of healthy individuals. The results of this triplicate experiment show an average increase of 1.6% when patients with high levels of TNF-﻿α are added to the sensor while decreasing by 3.5% when healthy serum is added as a negative control. Finally, through the proper definition of features, the impedance change of negative control and protein can be differentiated with the highest accuracy using a machine learning model (Fine Gaussian SVM).

## Supplementary Information

Below is the link to the electronic supplementary material.

Supplementary file1 (PDF 2.26 MB)

## Data Availability

No datasets were generated or analysed during the current study.
