# Supplementary material for: Wireless power-up and readout from a label-free biosensor
Source: Biomed Microdevices. 2025 Jan 10;27(1):2. doi: 10.1007/s10544-024-00728-9 (PMC11717847; doi:10.1007/s10544-024-00728-9)
Supplement: Supplementary file 1 — Supplementary file1 (PDF 2.26 MB) [file 10544_2024_728_MOESM1_ESM.pdf]

Etching step within the fabrication process:

After the fabrication of the bottom and top electrodes, as explained in Section 3.1 of the manuscript, several etching steps follow, as detailed in Fig. 1.

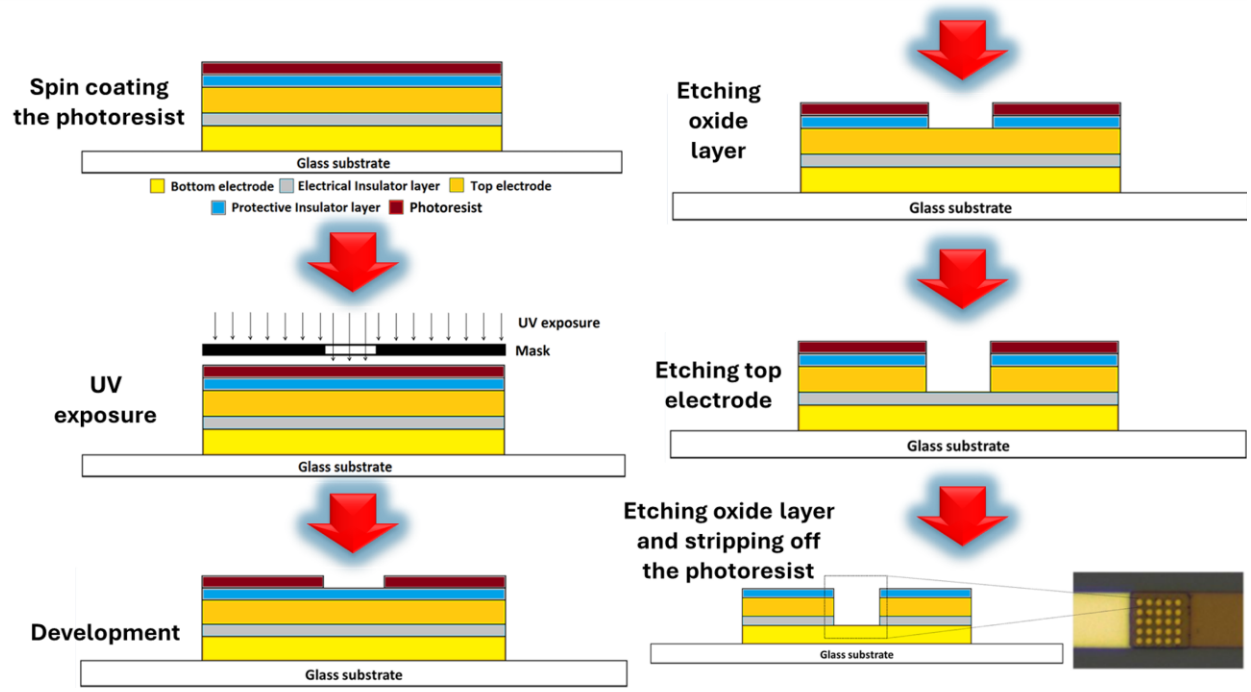

Figure 1. Etching step within the fabrication process.

The top left step in Fig. 1 shows different layers, including the fused silica substrate, bottom electrode, top electrode, and spin-coated photoresist used to pattern the well layer. The subsequent steps for etching include UV exposure, development, etching of the aluminum oxide layer, etching of the gold and chromium layers of the top electrode, and finally, etching of the aluminum oxide layer between the two electrodes.

Experimental setup configuration:

The sensing setup consists of two inductively coupled coils: a transmitter and a receiver, with the nanowell sensor connected in series on the receiver side alongside the receiver coil and a 26-ohm resistor. The transmitter side comprises a 500-ohm resistor, the transmitter coil, and a lock-in amplifier, all arranged in series. Both primary and secondary coils are wound in the same toroidal-shaped configuration with 35 turns each, using wire with a diameter of 0.6 mm. The following figure shows the different components of the setup (top view), with the transmitter coil on the left half of the toroidal shape and the receiver on the right half. The two coils have an angular separation of 0.454 radians between their axes. Using the average radius of the toroid (0.95 cm), the physical separation along the toroid's circumference can be calculated as r·θ, resulting in a distance of 0.43 cm.

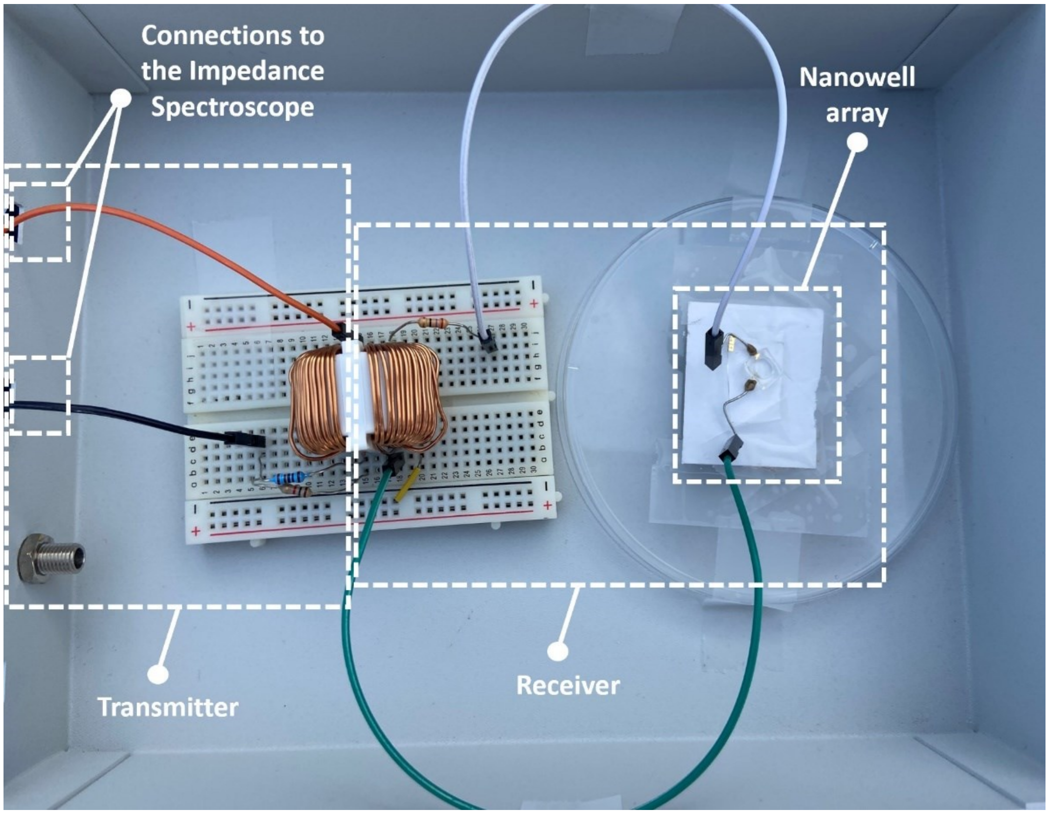

Figure 2. Experimental setup configuration.

Experiment procedure:

Different steps of the experimental procedure to detect the target protein are shown in the following figure, which are explained in section 4.1 of the manuscript.

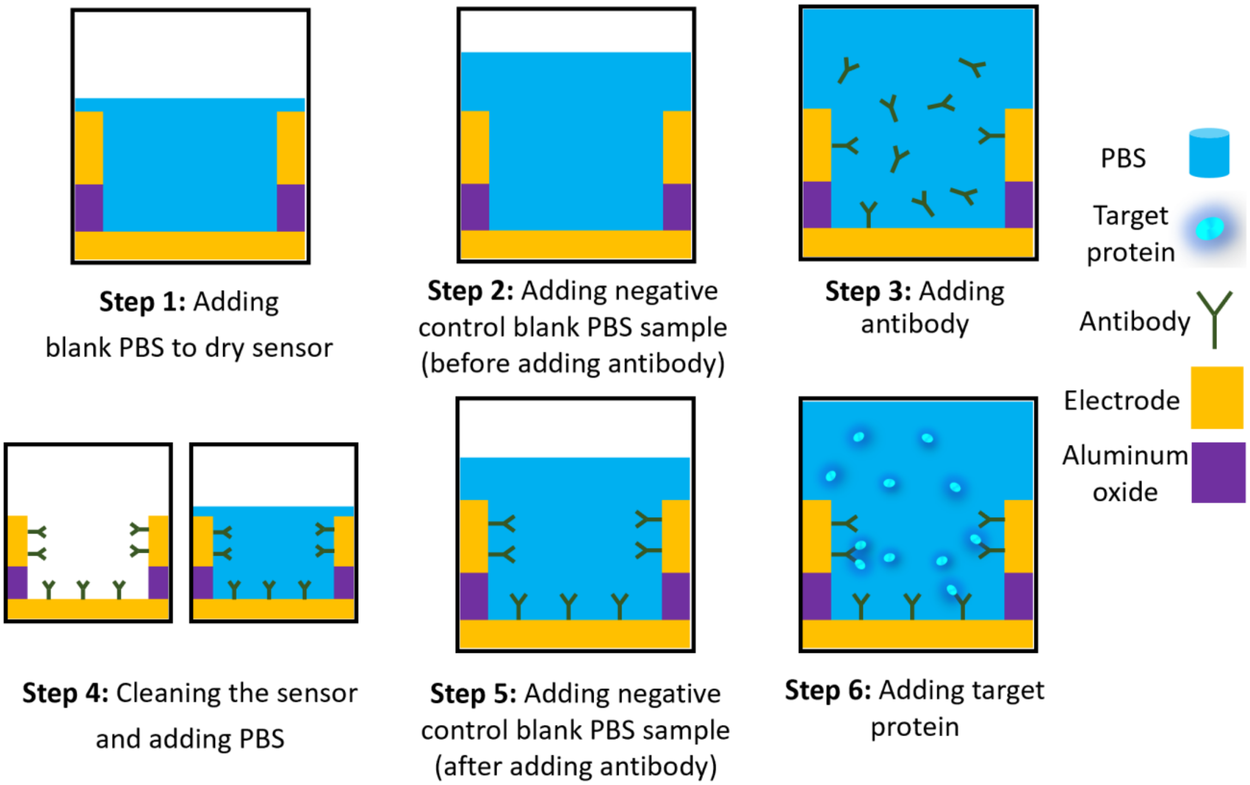

Figure 3. Different steps of the experiment procedure.

The phase component of the primary after adding PBS:

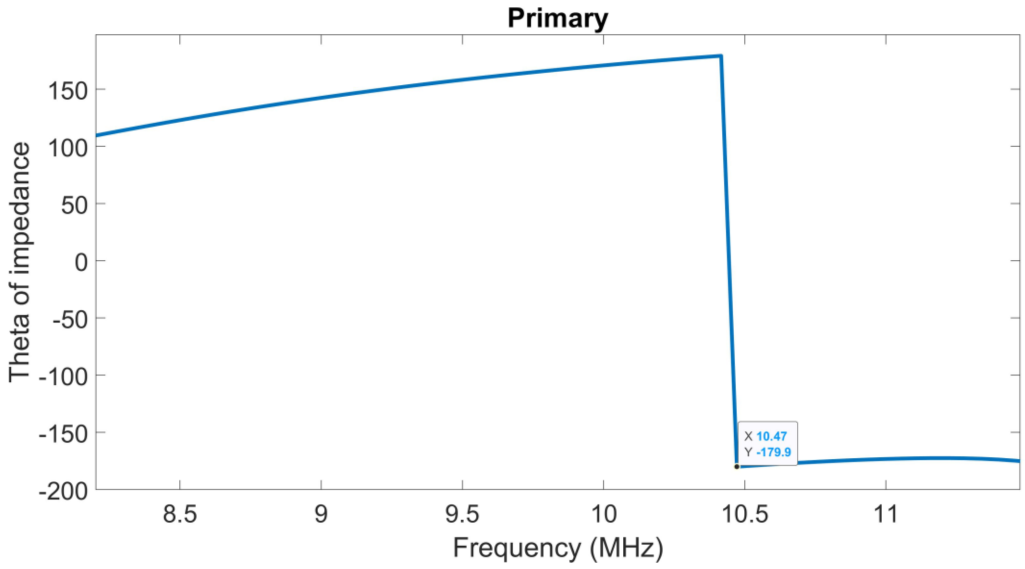

Figure 4. The phase component of the primary after adding PBS.

Comparison of the negative control response and HMGB1 in all the trials:

The results of triplicate experiments for the negative control (blank PBS sample) and the addition of the target, HMGB1, are as follows:

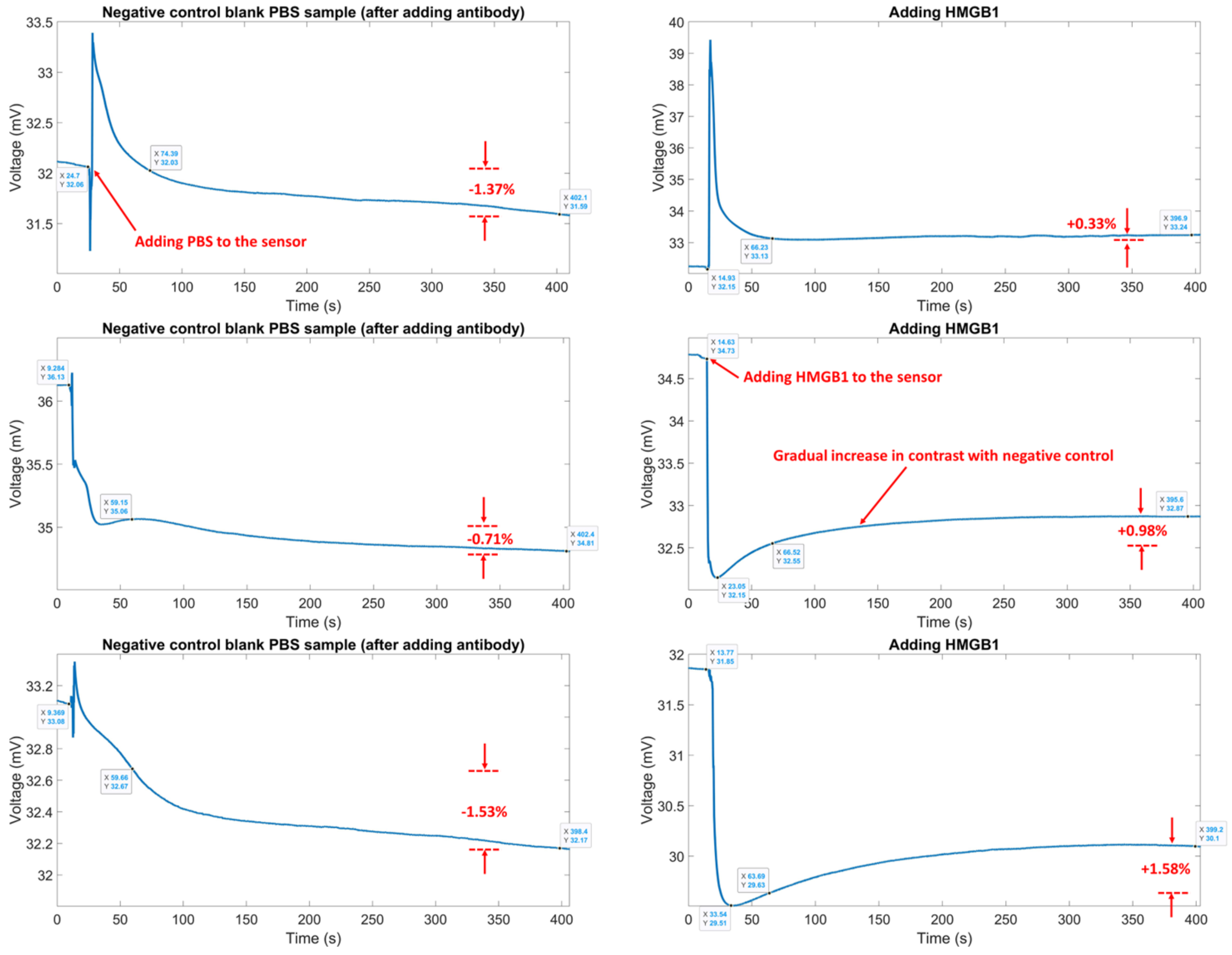

Figure 5. Comparison of the negative control response and HMGB1 across all trials. The left and right graphs show the negative control and HMGB1 responses, respectively.

Titration curve for CXCL-5 using nanowell array:

Extracting the titration curve provides important information on the sensor's response to sensitive measurements of various concentrations of this significant biomarker. Triplicate experiments are performed at several concentrations (3 nM, 300 pM, 30 pM, 3 pM, 300 fM) in a purified buffer using nanowell array. CXCL-5 serves as a biomarker in diseases like lung cancer and rheumatoid arthritis. These experiments are designed to validate sensor readings through impedance monitoring. The following figures show the titration curve for CXCL-5.

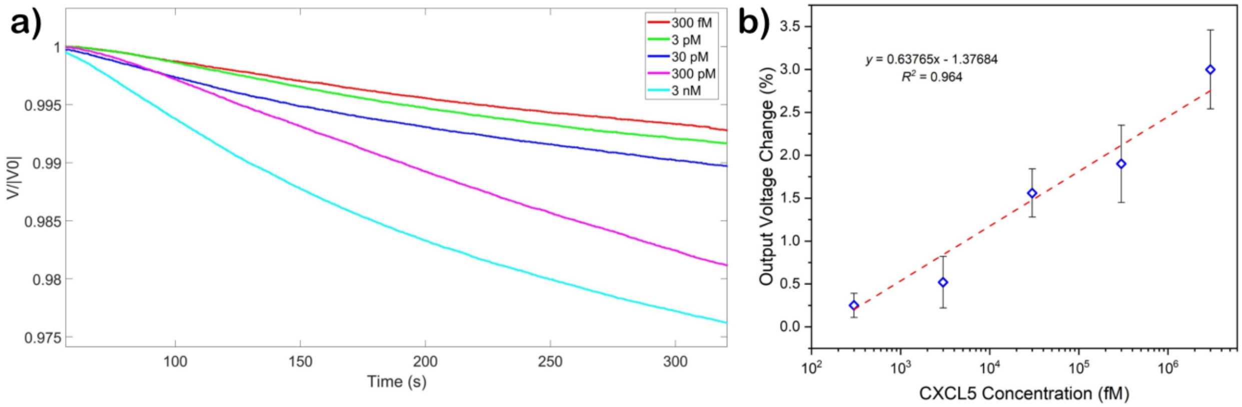

Figure 6. Titration curve for CXCL-5 a) Gradual decrease for different concentrations. b) Titration curve for CXCL-5.

The resulting titration curves for CXCL-5 highlight the nanowell array's capability to measure protein concentrations across a wide range accurately. These findings underscore the potential of this sensor technology in biomedical applications, where precise detection of biomarkers is crucial for disease diagnosis and monitoring. The experiments demonstrated a baseline shift in impedance plots with increasing CXCL-5 concentration. This gradual increase in impedance shift is attributed to the binding between CXCL-5 and its specific antibodies. At the lower concentration of 300 fM, the impedance shift closely resembles the responses observed in the negative control. This indicates that at such low concentrations, the sensor's ability to distinguish between the presence of CXCL-5 and the absence of the biomarker is limited. These findings highlight the sensor's sensitivity range and its effectiveness in detecting CXCL-5, particularly at higher concentrations where the impedance shift is more pronounced.
